# Recent Advances in the Treatment of Scedosporiosis and Fusariosis

**DOI:** 10.3390/jof4020073

**Published:** 2018-06-18

**Authors:** Matthew W. McCarthy, Aspasia Katragkou, Elias Iosifidis, Emmanuel Roilides, Thomas J. Walsh

**Affiliations:** 1 Division of General Internal Medicine, Weill Cornell Medicine of Cornell University, New York, NY 10065, USA; 2 Department of Pediatrics, Division of Pediatric Infectious Diseases, Nationwide Children’s Hospital and The Ohio State University, Columbus, OH 43205, USA; Aspasia.Katragkou@nationwidechildrens.org; 3 Infectious Diseases Section, 3rd Department of Pediatrics, Faculty of Medicine, Aristotle University School of Health Sciences, 54642 Thessaloniki, Greece; iosifidish@gmail.com (E.I.); roilides@med.auth.gr (E.R.); 4 Transplantation-Oncology Infectious Diseases Program, Departments of Pediatrics, and Microbiology & Immunology, Weill Cornell Medicine, New York, NY 10065, USA; thw2003@med.cornell.edu

**Keywords:** fusariosis, scedosporiosis, isavuconazole, voriconazole, MALDI-TOF

## Abstract

Species of *Scedosporium* and *Fusarium* are considered emerging opportunistic pathogens, causing invasive fungal diseases in humans that are known as scedosporiosis and fusariosis, respectively. These mold infections typically affect patients with immune impairment; however, cases have been reported in otherwise healthy individuals. Clinical manifestations vary considerably, ranging from isolated superficial infection to deep-seated invasive infection—affecting multiple organs—which is often lethal. While there have been a number of advances in the detection of these infections, including the use of polymerase chain reaction (PCR) and matrix-assisted laser desorption ionization/time-of-flight mass spectrometry (MALDI-TOF MS), diagnosis is often delayed, leading to substantial morbidity and mortality. Although the optimal therapy is controversial, there have also been notable advances in the treatment of these diseases, which often depend on a combination of antifungal therapy, reversal of immunosuppression, and in some cases, surgical resection. In this paper, we review these advances and examine how the management of scedosporiosis and fusariosis may change in the near future.

## 1. Introduction

The past few decades have witnessed a remarkable increase in the prevalence and severity of invasive fungal infections (IFI) in children and adults with immune impairment, including patients with hematologic malignancies, stem cell and solid organ transplantations, and primary and acquired immunodeficiencies and preterm neonates [1,2,3]. The majority of these infections are due to *Candida* spp. and *Aspergillus* spp., while less common fungal pathogens, such as mucormycetes, *Scedosporium* spp., and *Fusarium* spp. are reported with increasing frequency [4,5]. Diagnostic and therapeutic options against IFI have evolved considerably over the past several years, and in this paper, we will review how these advances affect the management of scedosporiosis and fusariosis in children and adults.

## 2. Scedosporiosis

### 2.1. Diagnosis

The timely diagnosis of mycotic disease is an enduring challenge in clinical practice. A diagnosis of *Scedosporium* spp. usually relies on the detection of fungi from clinical samples by direct microscopic examination or histological analysis of the clinical specimen and culture on appropriate culture media [6]. However, it may be difficult to distinguish *Scedosporium* spp. from species of *Fusarium* or *Aspergillus*, as all of them present dichotomous branching, hyaline hyphae, and regular hyphal septation [4,7]. Given this difficulty, newer approaches have been pursued, including non-culture-based molecular methods that utilize nucleic acid sequencing and mass spectroscopy.

Nucleotide sequence-based analysis is the current gold standard for fungal identification; rDNA internal transcribed spacer (ITS) sequencing appropriately identifies the main species in *Scedosporium*, but the partial β-tubulin gene (*BT2*) is required to differentiate closely related species [8,9]. Matrix-assisted laser desorption ionization/time-of-flight mass spectrometry (MALDI-TOF MS) has become available for the first-line identification of filamentous fungi, as its accuracy is comparable to that of DNA sequencing, but it is not routinely available at many medical centers [10].

Polymerase chain reaction (PCR)/electrospray ionization time-of-flight mass spectrometry (ESI-TOF-MS) combines 16 PCR assays using broad-range primers targeting nuclear or mitochondrial genes and T2 magnetic resonance (T2MR), which enables rapid determination of molecular weight and base composition in the amplicons after electrospray ionization and chromatographic separation [11,12,13]. This information may be compared to a database to identify fungal species, including *Scedosporium*, without the need for purification or extraction. However, this platform is expensive and is not routinely used at most centers; moreover, the database of filamentous fungi is relatively small, and the process of specimen preparation may be cumbersome [14,15,16]. These platforms highlight novel strategies to hasten the identification of *Scedosporium* spp., as a delay in diagnosis is often associated with substandard treatment and poor outcomes.

### 2.2. Clinical Manifestations

Species of the pathogenic mold *Scedosporium*, including *Lomentospora prolificans* (formerly *S. prolificans*), cause a wide range of clinical manifestations in humans from superficial infection to severe invasive disease, as well as colonization of the respiratory tract and allergic reactions [6,17]. Scedosporiosis is classically associated with hematological malignancies (HM), although patients with other forms of immune impairment may also contract the disease [18]. Given the variety of symptoms, clinicians should have a high index of suspicion in susceptible patients. Individuals suffering from near-drowning events in water are also at risk of disease, which may be associated with central nervous system (CNS) involvement [19]. Species of *Scedosporium*, including *S. boydii*, *S. apiospermum*, and *S. aurantiacum* are among the most commonly recovered molds from respiratory secretions of patients with chronic pulmonary diseases such as cystic fibrosis (CF) and may lead to invasive disease [4,7,20,21].

### 2.3. Treatment

There are three major classes of antifungal agents approved for use in humans: (1) triazoles (fluconazole, voriconazole, isavuconazole, itracontazole); (2) polyenes (various formulations of amphotericin B); and (3) echinocandins (casprofungin, micafungin, anidulafungin) [22,23,24,25,26,27]. The triazoles and polyenes have varying levels of activity against species of *Scedosporium*, with the azoles (such as itraconazole and voriconazole) typically having the lowest mean inhibitory concentration (MIC) among the antifungal drugs [28]. *Scedosporium* species are typically resistant to polyenes, as well as to fluconazole and demonstrate reduced susceptibility to echinocandins [29,30]. The high degrees of intrinsic antifungal resistance among species of *Scedosporium* make these infections difficult to manage and susceptibility testing of isolates from patients with scedosporiosis is highly recommended [21,31]. Reversal of immunosuppression is often crucial for successful management of infection [19].

Although the optimal choice and duration of therapy for scedosporiosis is controversial and may depend on the immune status of the patient, a large retrospective study provides support for the use of voriconazole [32]. In that study by Troke and colleagues, the most common underlying conditions were solid organ transplant (SOT, 22%), hematologic malignancy (HM, 21%), and trauma or surgery (15%) [29]. Two-thirds of patients with *S. apiospermum* infection had successful responses. Patients with malignancy and hematopoietic cell transplant (HSCT) recipients had the worst outcomes [29].

Most international guidelines recommend voriconazole as first-line therapy; however, antifungal combination therapy (ACT) has emerged as a promising option, because therapeutic effect can be achieved at lower concentrations thereby reducing toxic side effects, improving safety and tolerability, and shortening the therapeutic effect while potentially preventing treatment failure [33,34,35,36]. The combination of voriconazole with a polyene or an echinocandin has shown synergistic effects against both *S. apiospermum* and *L. prolificans*; however, these combinations have demonstrated variable outcome in the treatment of these infections in humans [18,37]. Combinations of three antifungal agents (voriconazole plus a polyene plus an echinocandin) have been tested against *L. prolificans* and showed synergy in vitro, but the data in humans with scedosporiosis is exceedingly limited.

In March 2015, the FDA approved the extended-spectrum triazole isavuconazole for the treatment of invasive aspergillosis and mucormycosis [38,39]. The advantages of this new antifungal drug include the availability of a water-soluble intravenous formulation, excellent bioavailability of the oral formulation, and predictable pharmacokinetics in adults [40,41]. Although this agent has not been approved for the treatment of scedosporiosis, in vitro data suggests that it may have good activity [42].

Guinea and collaborators examined the activity of isavuconazole against more than 1000 opportunistic fungi collected from 1986 to 2007, including 22 isolated of *Scedosporium* spp. [43]. The two species of *Scedosporium* tested (*S. prolificans* and *S. apiospermum*) showed marked differences in susceptibility: isavuconazole showed good activity against *Scedosporium apiospermum*, with a MIC_90_ very similar to those for *Aspergillus* spp., whereas *S. prolificans* (*L. prolificans*) was relatively resistant. Subsequent work by Pfaller and colleagues has reinforced this finding [44]. Indeed, isavuconazole demonstrates broad-spectrum activity against a global collection of opportunistic fungi; however, clinical experience with isavuconazole to treat scedosporiosis is limited, and its use is not routinely recommended.

The novel compound F901318 represents a new class of antifungal drug called the orotomides, which inhibits dihydroorotate dehydrogenase, a key enzyme in pyrimidine biosynthesis [45]. This drug has been evaluated for activity against 50 clinical *Scedosporium* and *Lomentospora* isolates [27]. F901318 displayed activity against all isolates *S. apiospermum, S. boydii*, and *S. aurantiacum*, with the MIC decrease ranging from 0.125 to 0.5 mg/L. Similarly, Wiederhold and collaborators had similar results with F901318 against *S. apiospermum, S. aurantiacum, S. dehoogii,* and *S. boydii*, with the MIC ranging from ≤0.008 to 0.25 [46]. Clinical trials are currently underway.

## 3. Fusariosis

### 3.1. Diagnosis

Although diagnosis may be suspected based on clinical phenomenon in high-risk patients, such as characteristic skin lesions in patients with acute myelogenous leukemia (AML), confirmatory diagnosis of fusariosis by histopathology and culture is strongly recommended by the European Confederation of Medical Mycology and the European Society of Clinical Microbiology and Infectious Diseases (ECMM/ESCMID) guidelines [47,48]. However, given the time and labor associated with these endeavors, other methods have been pursued.

Immunohistochemistry uses antibodies directed at cell antigens to identify fungal organisms visualized in situ. However, this approach is not widely used. Recently, DNA-based assays have emerged as a promising new approach to diagnosing fusariosis. A PCR platform based on the intergenic spacer (IGS) region has been developed for different *Fusarium* species that can also distinguish clinical species complexes, such as *Fusarium equiseti* and *F. sporotrichioides* [49]. A fluorescent PCR fragment length analysis based on the internally transcribed spacer 2 (ITS2) region is available to distinguish between *Aspergillus*, *Candida*, and *F. oxysporum*, but this method cannot distinguish between the *Fusarium* spp. that have closely-related ITS amplicons [50,51,52].

Multi-locus sequence typing (MLST) is currently viewed as the best option for the identification of *Fusarium* isolates on the species level, and enzyme-linked immunosorbent assay (ELISA) is the preferred method in *Fusarium* to identify specific mycotoxins [51,53,54,55]. As noted above, MALDI-TOF MS is a promising new tool for rapid identification and classification of cultured microorganisms based on their protein spectra [56,57,58]. However, the platform is not widely available and requires the recovery of the organism for processing. We anticipate that in the coming years, this methodology will become the preferred method for identification of fusariosis, especially at tertiary-care and academic medical centers.

### 3.2. Clinical Manifestations

*Fusarium* species cause a variety of diseases in humans, including superficial skin infections, keratitis, blood stream infections, and life-threatening end organ damage [59,60]. Infection may be fatal, as there is often a delay in diagnosis and many organisms exhibit high-levels of resistance to existing antifungal agents [61,62]. *Fusarium* spp. are a frequent cause of corneal damage and may lead to endophthalmitis [62]. Although onychomycosis as a result of fusariosis usually causes localized infection in immunocompetent patients, it may also represent the portal of entry for disseminated disease in patients with immune impairment [48,63].

Less commonly, *Fusarium* spp. may cause peritonitis, bloodstream infection, osteomyelitis (often after trauma), arthritis, otitis, sinusitis, and brain abscess [19,48]. As we will discuss below, treatment often involves antifungal therapy in conjunction with surgical resection and reversal of immunosuppression when possible.

### 3.3. Treatment

*Fusarium* spp. often display high levels of resistance to existing antifungal agents and are some of the more difficult fungi to treat [62,64,65]. Data on their in-vitro susceptibility to various antifungal agents indicate variable susceptibility amphotericin B and extended-spectrum triazoles, such as itraconazole, voriconazole, isavuconazole and posaconazole [51,53,54,66,67]. For this reason, we typically initiate empirical therapy with an antifungal triazole and a poleyene, such as voriconazole and liposomal amphotericin B, while awaiting antifungal susceptibilities. One notable exception to this approach may be for *F. solani*, which is somewhat more susceptible to amphotericin B but less susceptible to voriconazole than other species, such as *F. oxysporum* [68,69]. However, the optimal treatment for disseminated fusariosis has not been established. While polyenes and itraconazole have been associated with some success, voriconazole is the only agent with an indication for treating refractory fusariosis in the United States [19]. 

Essential for successful therapeutic outcome is the restoration of innate host defenses, particularly with recovery from neutropenia. GCSF may accelerate recovery from neutropenia. Granulocyte transfusions from GCSF-mobilized donors may stabilize infection until recovery from neutropenia in persistently neutropenic patients with hematological malignancies or in those with aplastic anemia. 

There are currently a number of novel antifungal agents in the pre-clinical pipeline that may have activity against human fusariosis [70]. These include E1210, a novel isoxazolyl bis-pyridine wall-active antifungal compound (discovered by the Eisai Company in Japan) that inhibits an early step in the GPI-dependent anchoring cell wall proteins and has in vitro activity against *Fusarium* spp. [71,72,73]. The pyrimidine salvage pathway offers another potential target (as noted above). F901318 is highly active in vitro against triazole-resistant mold pathogens, including *Scedosporium* and *Fusarium* species [70,74]. Genetic and biochemical analyses indicate that hemofungin may serve as yet another potential treatment option, as it inhibits ferrochelatase—the last enzyme in the heme biosynthetic pathway—and inhibits in vitro growth of pathogenic *Fusarium* species [70]. We are encouraged by this early work and are eager to see if these promising results may have utility in humans.

## 4. Strategies for Augmentation of Host Defenses against Scedosporiosis and Fusariosis

Host defenses against filamentous non-*Aspergillus* filamentous fungi are less well understood than those against aspergilli. However, limited data with *Fusarium* spp. and *Scedosporium* spp. show certain similarities in pathogenesis and host defenses with *Aspergillus fumigatus,* with which most studies have been performed.

The predominant line of host defenses against filamentous fungi is circulating polymorphonuclear (PMNs) and mononuclear leukocytes (MNCs), as well as monocyte-derived macrophages (MDMs) [75]. MDMs in the lungs and elsewhere phagocytize and destroy spores of difficult-to-treat filamentous fungi, such as *S. apiospermun* and *S. prolificans* [76,77], comparably to *A. fumigatus*. If the immune response of the host is compromised, spores can germinate to hyphae and invade to adjacent tissues. PMNs are the main immune cells causing damage to hyphae using oxygen-dependent (O_2_^−^, H_2_O_2_, hypohalides, and chloramines) and oxygen-independent (cationic peptides such as defensins and cathelicidins) mechanisms [78]. Phagocytes are capable of exhibiting sufficient oxidative burst to control *S. prolificans* [77].

A variety of growth factors, cytokines, and chemokines play an important role in the interface of innate and adaptive immunities against filamentous fungal infections [79]. However, very little is known about how fungal elements are recognized by macrophages and how the signal is transduced to the nucleus for gene expression and release of cytokines in response to *Fusarium* or *Scedosporium* spp. Of note, *S. prolificans* has been shown to induce significantly more TNF-α and IL-6 release by human MNCs as compared to *Aspergillus* spp., which could be associated with the virulence of the specific fungus [80]. Differences in immune response and damage of different genera of filamentous fungi and indeed of different species of *Fusarium* and *Scedosporium* are likely linked to the frequency and severity of infections by some of these fungi.

A number of studies have assessed the immunomodulatory utility of cytokines in confronting fungal pathogens. Th1- and Th17-type cytokines have exhibited certain enhancing activities on antifungal phagocytic responses. For example, IL-15 increased interleukin-8 (IL-8) release from PMNs challenged by *F. solani* hyphae, but not by *F. oxysporum* hyphae. In contrast, the release of TNF-α was not affected by the use of IL-15 [81]. Similarly, IL-15 increased IL-8 release from PMNs challenged by *S. prolificans,* whereas release of TNF-α was not affected. In addition, the presence of IL-15 significantly enhanced PMN-induced hyphal damage and oxidative respiratory burst of *S. prolificans* but not *S. apiospermum.* This inability of IL-15 to exhibit enhanced damage of *S. apiospermum* hyphae is in concordance with this fungus’ greatest intrinsic virulence in humans. IL-15 may have species-specific enhancing effects on antifungal activities of PMNs against *Scedosporium* spp. and *Fusarium* spp. [81].

Among other cytokines studied that enhance PMN antifungal activity against *Scedosporium* spp. are interferon-γ (IFN-γ) and granulocyte-macrophage colony-stimulation factor (GM-CSF) [82]. These cytokines induce the Th1 response, which favors resistance to fungal disease, regulates the gene expression of NADPH oxidase subunits at the transcriptional level, and potentiates the synthesis of antimicrobial peptides in macrophages [83]. GM-CSF acts on early as well as on late stages of haematopoiesis and increases the number of cells of the macrophage–monocyte system. It has been found to enhance phagocytosis, oxidative burst, increase the number and membrane binding of several classes of surface receptors on PMNs, and inhibit PMN apoptosis [84,85]. Treatment of PMNs with the combination of IFN-γ and GM-CSF had broader effects on *Scedosporium* spp., enhancing PMN functions including oxidative burst in response to *S. apiospermum* hyphae [82].

Antifungal agents may also have differential immunomodulatory effects against *Fusarium* and *Scedosporium* spp. In vitro studies have shown that MDM activity against *Fusarium* spores [86], oxidative antifungal activities of human MNCs and PMNs against *F. solani* hyphae [87], and PMN activity against *S. prolificans* and *S. apiospermum* [88] can be modulated by the presence of different amphotericin B formulations. Similarly, triazoles have caused significant additive increase in PMN hyphal damage of *S. prolificans* and *S. apiospermum* [89]. Regardless of the mechanisms behind these collaborative effects, these findings support the concomitant administration of antifungals and PMN transfusions to persistently neutropenic patients with invasive fusariosis or scedosporiosis.

No animal model studies have reported on the effects of cytokines, such as granulocyte colony stimulating factor (G-CSF), on the outcome of experimental fusariosis to date. Ortoneda et al., in an immunosuppressed murine model of invasive infection by *S. prolificans*, demonstrated a modest efficacy of liposomal amphotericin B (LAMB) at 10 mg/kg/day combined with G-CSF [90]. Subsequent studies showed that LAMB at very high doses (40 mg/kg/day) combined with G-CSF did not significantly improve survival [91]. Interestingly, the administration of G-CSF alone was not more effective as compared to the control group [90,91]. In an immunocompetent murine model of disseminated *S. prolificans* infection, posaconazole and GM-CSF had a combined effect in damaging *S. prolificans* hyphae*.* However, when posaconazole and GM-CSF were administered to mice with invasive infection due to *S. prolificans*, they had selective beneficial effects on the burdens in certain organs but offered no additional benefit to survival [92].

The principal therapy in fusariosis is early aggressive antifungal therapy. Immunomodulation with G-CSF or GM-CSF potentially combined with IFN-γ can be adjuvant therapeutic strategies together with source control. Over the last decades, efforts to reconstitute host defenses with granulocyte transfusion therapy have increased after the advances of the availability of recombinant hematopoietic growth factors and modern transfusion practices. In severely neutropenic patients suffering from fusariosis, treatment with G-CSF or GM-CSF and granulocyte transfusions may be considered [93,94,95,96,97]. The beneficial effect of the granulocyte transfusions seems to be enhanced when they are collected after stimulation of donors with G-CSF and dexamethazone and are administered to patients with good performance status, as well as early during neutropenia and soon after the onset of fungal infection [98]. In a systematic review of 23 cases with invasive fusariosis, including eight patients aged less than 18 years, granulocyte transfusion using traditional collection protocols failed to augment the efficacy of appropriate antifungal treatment. In contrast, in a case series of 11 invasive fusariosis from a single center, remarkably high response and survival rates were found when antifungal agents were combined with granulocyte transfusions, which were collected using a specific donor collection protocol (donors were stimulated by G-CSF and dexamethasone).

The clinical evidence on the role of immunomodulation on the host response against scedosporiosis is limited, involving mainly haematopoietic growth factors (G-CSF or GM-CSF) and IFN-γ [99]. Of note, of 39 cases reported with disseminated *Scedosporium* spp. infection, a favorable outcome was reported only in four of them [100]. In these four cases, the positive outcome was attributed to the immunomodulatory factors administered in addition to antifungal drugs, whereas the true role of antifungals was difficult to establish [101,102]. Without doubt, the outcome of antifungal drug therapy alone in scedosporiosis is poor with high overall mortality. A crucial point in the management of this difficult-to-treat infection is immune function reconstitution. Characteristically, only 2 out of 16 patients infected with *S. prolificans* survived, and their survival coincided with hematologic recovery [103]. Similarly, in another review, disseminated *S. prolificans* infection was fatal in all neutropenic patients [104].

## 5. Management of *Scedosporium* and *Fusarium* Infections in Children

Pediatric pharmacokinetic, safety, and efficacy data are sparser than those in adult patients. As a consequence, the pace of guideline development for pediatric patients has been much slower than that for adults, and indeed, dedicated guidelines for treating infants and children with IFI do not exist. Extrapolating adult recommendations to pediatric practice should be done cautiously, as differences pertaining to epidemiological factors and pharmacokinetics of many antifungal agents exist between adults and children.

### 5.1. Pediatric Scedosporiosis

*Scedosporium* spp. infections in children have been associated with brain abscess formation after near drowning in immunocompetent individuals [19]. These infections are inherently difficult to treat given the delayed diagnosis, the histologic similarities of spedosporium infection with other more frequently encountered filamentous fungi, the cross reactivity of immune reactions with other fungi, and the false negative immune reaction results due to the high genetic variability of *Pseudallescheria* spp. [105]. The most recent reports with favorable outcomes indicate that voriconazole may be an effective antifungal agent, as salvage treatment options, parenteral administration of terbinafine and voriconazole in addition to intraventricular caspofungin, and neurosurgical interventions have been used but with mixed results [106]. Among adjunctive therapeutic modalities, the administration of certain cytokines interferon (IFN-γ) and granulocyte-macrophage colony-stimulation factor (GM-CSF), which induce an augmentation of neutrophil antifungal activity against *Scedosporium* spp., has been proposed [82].

### 5.2. Pediatric Fusariosis

*Fusarium* spp. infections are between the third and fourth most common cause of IFI in children; *F. solani* and *F. oxysporum* species complex are the most commonly implicated in pediatric disease [107,108,109]*.* Clinical presentation of *Fusarium* spp. infections depends on the portal of entry, as well as the host’s immune status. In immunocompetent patients, infections are usually superficial (onychomycosis, keratitis, or mycetoma) or limited to a single organ (lungs or paranasal sinuses), whereas in immunosuppressed hosts, such as allogeneic hematopoietic stem cell recipients, patients with severe or prolonged neutropenia, or acute leukemia, fusariosis may be invasive and disseminated [63,110].

Given the rarity of these infections, treatment guidelines are not based on randomized controlled clinical trials but on large uncontrolled case series mainly in adult patients [111]. Especially in the pediatric population, treatment of scedosporiosis and fusariosis is challenging, with all-cause mortality in pediatric case series as high as 50% [108,112]. Evidence-based data for guiding treatment are scant, and usually treatment recommendations are inferred from adult experience. On many occasions, salvage treatment options are required, which include the use of newer antifungal agents and practices aiming to reduce immunosuppression [111,112].

The FungiScope registry, a global fungal infection registry, found 10 children with invasive fusariosis between 2006 and 2015 [113]. All these patients were immunosuppressed and neutropenic, and among these, 80% received combination therapy with voriconazole and either a lipid formulation of amphotericin B (50%) or an echinocandin (30%), and only 20% received voriconazole monotherapy. Surgery was performed in 30%, granulocyte transfusion and granulocyte colony stimulating factor (G-CSF) were used in 40% of the patients each.

A recent literature review of invasive *Fusarium* infections in immunocompromised children (including five new cases, summarizing 33 cases in total) showed considerable variation in the treatment regimens used: Amphotericin B was used in most cases (76%); combination treatment with (amphotericin B and voriconazole; amphotericin B and caspofungin; amphotericin B, fluconazole, and rifampin; amphotericin B and ketoconazole; amphotericin B, 5-FC, and rifampin) was used in 33%; G-CSF was used in 18%; granulocyte transfusion in 12%; and surgery was performed in 12% of the cases [108]. In both of the above studies, a favorable outcome was noted in 50% of the patients [108,112].

Posaconazole has been recommended as a salvage treatment option for invasive fusariosis in high-risk patients with hematologic malignancies [111]. Data for posaconazole use for fusariosis treatment come from three open label clinical trials [114] and a retrospective single institution analysis [115], which hardly included any children (<18 years of age). The limited use of posaconazole in children could be due to limited experience in patients less than 12 years of age [111,112] (Table 1). Similarly, there is no experience with isavuconazole use in children with fusariosis [116]. The two clinical trials (SECURE and VITAL), which evaluate the use of isavuconazole in the treatment of fungal infections, including rare fungi, enrolled patients who were more than 18 years old. Thus far, they have reported only seven patients with fusariosis treated with isavuconazole, resulting in a 44% survival rate [117].

Data for combination antifungal therapy in children with IFI, including invasive fusariosis, are scant; however, it is frequently used in pediatrics as evidenced by recent pediatric cohort studies [107,109,118]. Of note, the association between the receipt of combination therapy and an improved outcome has not been found. The predilection for clinicians to use combination antifungal treatment for invasive fusariosis is probably due to high concern for high or intrinsic resistance of *Fusarium* spp., the severity of the infection, high mortality—especially in patients with multiple comorbidities requiring salvage treatments—and their uncertainty about the effectiveness and options in treating this serious infection.

A promising approach for the fungal strains that are resistant to currently used antifungal agents or for patients with compromised host immunity is immune based treatments. While, until now, most immunotherapeutic approaches have aimed to augment the number of granulocytes through granulocyte transfusions, the infusion of growth factors (G-CSF, GM-CSF), the administration of cytokines such as IFN-γ, and most recently, the use of adoptive T cell therapy, which was initiated for the treatment of cancer, seems to be a promising approach for the treatment of patients suffering from drug-resistant IFI [119]. Even though there is growing evidence supporting the role of T cell adoptive therapy in antifungal immunity, the clinical development of fungus-specific T cells is in the early stages of development, and there is a paucity of data regarding adoptive T cell therapy using *Scedosporium-* or *Fusarium-*specific T cells [120].

In summary, the optimal treatment for scedosporiosis and fusariosis in children is unknown. Voriconazole demonstrates strong in vitro activity against *Scedosporium* spp and is considered first-line treatment. For fusariosis treatment, voriconazole, lipid formulations of amphotericin B, and various combinations should be considered as the optimal alternatives. Duration of treatment is usually individualized based on the site, the extent of the infection, and the immune status of the patient [63]. In addition, the optimal management should include surgical debridement of infected tissues and reinforcement of immune response either by reducing immunosuppression or augmenting immune response with the use of various growth factors or adoptive T cell therapies. However, the latter strategy is in its infant state of clinical development with unknown safety and efficacy outcomes.

## 6. Conclusions

Human scedosporiosis and fusariosis are emerging opportunistic infections that are difficult to diagnose and may be even more challenging to treat. In this paper, we have reviewed the challenges associated with diagnosis, which typically relies on labor-intensive histopathology, as well as recent advances in non-culture-based systems. We have also reviewed novel antifungal compounds, such as E1210, F901318, and hemafungin, which may one day play a role in treatment. However, these agents are still in development. For now, treatment of these two potentially-lethal conditions relies on early diagnosis, effective antifungal therapy, possible surgical excision, and reversal of immunosuppression when possible. In the coming years, the mycology community must make it a priority to design non-inferiority trials to evaluate these new agents to meet the needs of vulnerable patients.

## Figures and Tables

**Table 1:** Pediatric doses of systemic antifungal agents.

Agent	Daily Dosage Per Age Group				
	>18 Years	13–18 Years	2–12 Years	1–24 Months	Neonates
Amphotericin B deoxycholate	1–1.5 mg/kg QD	1–1.5 mg/kg QD	1–1.5 mg/kg QD	1–1.5 mg/kg QD	1–1.5 mg/kg QD
Liposomal amphotericin B	3–5 mg/kg QD	3–5 mg/kg QD	3–5 mg/kg QD	3–5 mg/kg QD	3–5 mg/kg QD
Amphotericin B lipid complex	5 mg/kg QD	5 mg/kg QD	5 mg/kg QD	5 mg/kg QD	5 mg/kg QD
Amphotericin B colloidal dispersion	3–4 mg/kg QD	3–4 mg/kg QD	3–4 mg/kg QD	3–4 mg/kg QD	n/a
Itraconazole IV	200 mg BID (for 2 days), followed by 200 mg QD	n/a	n/a	n/a	n/a
Itraconazole oral suspension/capsules*	600 mg QD (for 3 days), followed 400 mg QD	2.5 mg/kg BID	2.5 mg/kg BID	n/a	n/a
Voriconazole IV*	6 mg/kg Q12h on day 1 then 4 mg/kg BID	4 mg/kg BID	8 mg/kg BID	n/a	n/a
Voriconazole oral suspension/capsules*	200 mg BID	200 mg BID	9 mg/kg BID (max: 350 mg BID)	n/a	n/a
Posaconazole*	200 mg QID or 400 mg BID	200 mg QID or 400 mg BID	n/a	n/a	n/a
Caspofungin	50 mg/day (day 1: 70 mg)	50 mg/m^2^ (day 1: 70, max 70 mg)	50 mg/m^2^ (day 1: 70, max 70 mg)	50 mg/m^2^ (day 1: 70, max 70 mg)	25 mg/m^2^
Anidulafungin	100 mg/day (day 1: 200 mg)				
Micafungin	100 mg/day	100 mg/ m^2^	>40 kg: 100 mg/day <40 kg: 2–4 mg/kg/day	>40 kg: 100 mg/day <40 kg: –4 mg/kg/day	>40 kg: 100 mg/day <40 kg: –4 mg/kg/day

QD: once per day; BID: twice per day; QID: four times per day; IV: intravenous; PO: oral; n/a: not sufficient data. * Therapeutic drug monitoring is recommended. Adapted from [111].
